# Use and Outcomes of Sugammadex for Neurological Examination after Neuromuscular Blockade in the Emergency Department

**DOI:** 10.5811/westjem.29328

**Published:** 2025-01-30

**Authors:** Stephen D. Hallisey, Christiana K. Prucnal, Annette M. Ilg, Raghu R. Seethala, Paul S. Jansson

**Affiliations:** * Brigham and Women’s Hospital, Department of Emergency Medicine, Division of Emergency Critical Care Medicine, Boston, Massachusetts; † Harvard Medical School, Department of Emergency Medicine, Boston, Massachusetts; ‡ Brigham and Women’s Hospital, Department of Emergency Medicine, Boston, Massachusetts; § Massachusetts General Hospital, Department of Emergency Medicine, Boston, Massachusetts

## Abstract

**Introduction:**

Non-depolarizing agents such as rocuronium and vecuronium are frequently used in the emergency department (ED) to facilitate intubation but may lead to delay in neurologic examination and intervention. Sugammadex is used for reversal of neuromuscular blockade by non-depolarizing agents but its role in the reversal of neuromuscular blockade for neurologic examination in the ED is poorly defined.

**Methods:**

This was a multicenter cohort study using retrospective chart review. We reviewed all ED encounters from June 21, 2016–February 9, 2024 of the electronic health record of Mass General Brigham, a large multistate health system, and abstracted all ED administrations of sugammadex to facilitate neurologic examination. We calculated descriptive statistics and assessed outcomes.

**Results:**

In 3,080,338 ED visits during the study period, 48 patients received sugammadex to facilitate neurologic examination. Of those patients, 23 (47.9%) underwent a procedure within 24 hours. Three (6.3%) had bradycardia, and one (2.1%) had hypotension following sugammadex administration. A total of 23 patients (47.9%) ultimately died during their admission, and 24 (50%) died within 30 days.

**Conclusion:**

Patients who received sugammadex in the ED to facilitate neurologic examination during the study period had rare associated adverse effects, high rates of procedures within 24 hours of administration, and significant in-hospital mortality. Prospective data is needed to assess the impact of sugammadex on decision-making.

Population Health Research Capsule What do we already know about this issue?

*Little is known about the use of sugammadex in the ED. Prior data has shown it is most commonly used to facilitate neurologic exam.*

What was the research question?

*What are the outcomes of patients who receive sugammadex for neurologic exam in the ED?*

What was the major finding of the study?

*47.9% of patients who received sugammadex in the ED ultimately underwent a procedure within 24 hours, and 50% died within 30 days.*

How does this improve population health?

*This study provides input on the outcomes of patients receiving sugammadex in the ED for neurologic exam, which is done rarely and in high-acuity and time-sensitive clinical situations.*

## INTRODUCTION

Neuromuscular blockade (NMB) is frequently administered as part of rapid sequence induction in the emergency department (ED) and prehospital settings. Non-depolarizing aminosteroid neuromuscular blocking agents (NMBA) such as rocuronium and vecuronium are commonly administered to facilitate intubation in the ED,^1^ but administration may lead to prolonged paralysis and delay in neurologic examination and surgical decision-making in patients presenting with neurological injury. Sugammadex is a modified gamma-cyclodextrin used for the reversal of NMB from aminosteroid NMBAs.^2^ When compared to acetylcholinesterase inhibitors such as neostigmine, sugammadex is associated with faster time to reversal, longer duration of action, and lower rates of cholinergic side effects such as bradycardia, nausea, and vomiting.^3^ As a result, it does not require the co-administration of atropine or glycopyrrolate. It is currently recommended over neostigmine as a first-line agent for the reversal of rocuronium in the operating room.^4^ Despite this recommendation, little is known about the use of sugammadex in the ED.

Two recent studies have highlighted the potential for its use in the ED setting.^5^ ^,^ ^6^ Our recent review of the use of sugammadex in the ED found that the most common indication was for neurologic examination, with 93.7% of patients receiving sugammadex for this indication.^7^ In that series, we found that the use of sugammadex for other indications was rare: one patient received sugammadex after inadvertent NMB administration; one received sugammadex to facilitate terminal extubation; and one received sugammadex following incomplete reversal of NMB at an ambulatory surgery center. No patients received sugammadex for a cannot-intubate-cannot-ventilate scenario. Only two small studies have described the use of sugammadex to facilitate neurological examination. A retrospective study of 11 patients receiving sugammadex in the ED for neurological examination found that the majority of patients who received sugammadex had a change in their examination and concluded its administration to be useful.^8^ A second retrospective study that evaluated its use in 24 patients found that dosing of 2 milligrams per kilogram (mg/kg) and 4 mg/kg were equally effective in achieving a train of four (TOF) of four.^9^ We sought to further define the safety, efficacy, and outcomes of sugammadex use to facilitate neurologic examination in the ED.

## METHODS

This was a multicenter cohort study using retrospective chart review. We adhered to all elements of optimal retrospective chart review in emergency medicine research as previously defined by Worster et al with the exception of interobserver reliability use and testing, as chart abstraction was performed by one author.^10^ We performed a structured chart abstraction of all ED encounters between June 21, 2016 (the date of sugammadex addition to the formulary) and February 9, 2024, in the electronic health record (EHR) (Epic Systems, Verona, WI) of Mass General Brigham, a large multistate regional health system with two affiliated academic medical centers and seven affiliated community, acute care hospitals.

We used chart abstraction to identify all administrations of sugammadex during an ED encounter. Charts were manually reviewed by the senior author (PSJ) to verify usage of sugammadex to facilitate neurological examination. Patient demographics, dosing weight, and laboratory values were automatically abstracted from the EHR for the linked ED encounter. For NMBAs administered within the health system, dosing weight, dose administered, and time of administration were automatically abstracted from the linked record of time of medication administration in the EHR. For NMBAs administered outside of the health system, linked EHRs, triage notes, and ambulance run reports were manually reviewed to determine dose and timing of NMB administration.

Manual chart review and abstraction was then performed by the first author (SDH) to determine the neurological injury type, procedure type and timing, major adverse events, mortality, and in-hospital changes to a comfort-oriented code status.

Where possible, Glasgow Coma Scale (GCS) was abstracted from nursing and physician notes. We calculated a modified Rankin Scale (mRS) based on the physical examination and physical therapy notes included in the discharge summary. Descriptive statistics were calculated. This research was approved by the Mass General Brigham institutional review board.

## RESULTS

### Patient Demographics

From June 21, 2016–February 9, 2024, there were 3,080,338 ED visits at Mass General Brigham-affiliated acute-care hospitals. Forty-eight patients received sugammadex to facilitate neurological examination. The mean (±SD) age at administration was 59.9 (±20.9) years of age (range 21–94 years). Sixteen patients (33.3%) were female, and 32 patients were male (66.7%) (Table).

**Table.** Patient characteristics.

Age, mean (SD), years	59.9 (20.89)
Sex, n (%)	
Male	32 (66.7%)
Female	16 (33.3%)
Weight, mean (SD) kg	77.3 (18.9)
Sugammadex dose, mean (SD), mg	345.63 (200)
Sugammadex dose, mean (SD), mg/kg	4 (2.8)
Neurologic injury, n (%)	
Subdural hematoma	6 (12.5%)
Subarachnoid hemorrhage	7 (14.6%)
Multicompartmental hemorrhage	15 (31.3%)
Intraparenchymal hemorrhage	11 (22.9%)
Ischemic stroke	3 (6.3%)
Cervical spine injury	3 (6.3%)
Other	3 (6.3%)
Trauma, n (%)	25 (52%)
Mortality, n (%)	
Within 72 hours of sugammadex	12 (25%)
Within 30 days of sugammadex	24 (50%)
Change to comfort measures only, n (%)	
Within 72 hours of sugammadex	16 (33.3%)
During hospitalization	22 (45.8%)
Location of paralytic, n (%)	
Outside hospital	9 (18.8%)
Prehospital	18 (37.5%)
Interhospital transfer	2 (4.2%)
Emergency department	19 (39.6%)
GCS, median, when recorded	
Pre-sugammadex	3
Post-sugammadex	4
Change pre-/post-sugammadex	4
Procedure performed, n (%)^*^	
Craniotomy	10 (20.8%)
External ventricular drain	7 (14.6%)
Angiogram/embolization/thrombectomy	4 (8.3%)
Spinal fusion/decompression	3 (6.3%)
Other	5 (10.4%)

* Some patients had more than one procedure.

*GCS*, Glasgow Coma Score; *kg*, kilogram; *mg*, milligram.

### Dosing, Timing, and Location

The mean dosing weight (±SD) was 77.3 kgs (±18.9 kg). Rocuronium was the most common NMBA to be reversed, used in 46 of the 48 patients (95.8%). We were able to abstract accurate dosing of rocuronium for 35 patients, and the mean (±SD) dose was 104.7 mg (±18.3 mg) or a mean of 1.37 mg/kg (±0.30 mg/kg). Accurate dosing of vecuronium was obtained from one patient, who received 10 mg (0.13 mg/kg).

Sugammadex was given at a mean dose of 346 mg (range 100–2,000 mg, interquartile range [IQR] 200–377.5 mg). The most common dose was 4 mg/kg (25 patients) with a mean dose of 4 mg/kg (range 2–18 mg/kg, IQR 2.8–4 mg/kg). The NMB and sugammadex were administered in the same ED encounter for 15 patients. For NMB given in alternate contexts, the most common location was prehospital (18 patients), at a referring hospital (13 patients), and during interfacility transport (two patients). All doses of sugammadex were administered in the two academic medical center EDs. We were able to obtain accurate time of administration for both NMB and sugammadex for 22 patients. The mean (range, ±SD) time from NMB to sugammadex administration was 109.9 (31–283, ±66.8) minutes.

With regard to hepatic and renal clearance, all patients had a creatinine level obtained during the ED visit, and 46 of 48 (96%) had transaminase levels obtained. Thirty-three patients had normal transaminase levels (defined as both aspartate aminotransferase and alanine aminotransferase < 50 units per liter) and 35 patients had normal creatinine levels (defined as < 1.2 mg/deciliter).

### Neurological Injury

The primary neurological injury varied, but the majority (40, 83.3%) of patients presented with intracranial hemorrhage. Three (6.3%) presented with acute ischemic strokes; two patients (4.2%) had extracranial vascular injury; one with a Type A aortic dissection causing common carotid artery occlusion and one with a vertebral artery dissection and pseudoaneurysm. Two (4.2%) patients had primary spinal cord trauma, and one patient (2.1%) presented with a brain mass. Twenty-five (52.1%) of the patients presented following trauma.

### Outcomes

An accurate GCS was obtained before administration of sugammadex in 43 (89.6%) patients and was 3t in all but one (who was 4t). An accurate GCS was obtained after administration of sugammadex in 35 (72.9%) patients with a mean (±SD) of 6.4 (±2.4). Thirty-four (70.8%) had a reliable GCS obtained before and after administration of sugammadex; the mean (range, ±SD) increase in GCS was 3.38 (−1 to +8, ±2.5) points.

Twenty-three patients (47.9%) underwent an invasive procedure within 24 hours of sugammadex administration, and another three (total of 26 or 54.2%) underwent a procedure within 72 hours (Table 1). Twelve patients (25.0%) died within 72 hours of sugammadex administration, while 24 patients (50%) died within 30 days. The code status was changed to “comfort measures only” for 16 patients (33.3%) within 72 hours of sugammadex administration, and for 22 patients (45.8%) during the hospitalization. Adverse events were rare, with two (4.1%) patients experiencing hypotension after sugammadex administration, four (8.2%) patients experiencing bradycardia, and no patients experiencing cardiac arrest.

The mRS for neurologic disability^11^ ^,^ ^12^ at discharge (ranging from 0–6, with higher scores indicating more severe disability), excluding all patients with a discharge mRS of 6 (deceased), was an average of 3.9 (SD ±1.36), where a score of 4 indicates moderately severe disability.

## DISCUSSION

Non-depolarizing NMBAs are frequently used in both the ED and prehospital setting during airway management of neurologically injured patients. The use of non-depolarizing NMBAs leads to prolonged paralysis, which impairs accurate neurologic examination essential to guide emergent and time-sensitive therapy for neurologic injury. Beyond neurologic examination being a critical part of decision-making regarding therapy, prognosis related to initial neurologic examination may be valuable to families as they consider early goals of care. This is reflected in our dataset as 33.3% of patients receiving sugammadex whose status was changed to “comfort measures only” within 72 hours of receiving sugammadex.

Rocuronium, the most commonly used NMBA in our cohort, has an expected duration of action of 30–60 minutes.^13^ However, longer duration of action has been well described.^14^ ^–^ ^17^ The time to administration of sugammadex in our study reflects this, as patients received sugammadex as long as 283 minutes following rocuronium administration with change in neurologic examination. Additionally, we were unable to obtain accurate times for some prehospital and referring hospital administrations of neuromuscular blockade, which may have biased the results toward those administered in the same ED encounter. This potentially extended duration of action for NMB was unlikely due to impairments of renal or hepatic metabolism as these was predominantly normal in our cohort; instead, it may have been due to higher NMB doses used, greater patient age, or to uncharacterized hypothermia or hypovolemia, the latter of which was not captured in our study.^18^ ^–^ ^20^ All doses of sugammadex occurred at the two academic medical centers. We attributed this to both hospitals being referral centers for neurosurgical trauma and for post-stroke care. Because of this, we are unable to draw any conclusions about its use in community hospitals.

Our study replicated previous findings seen in the relevant literature including that sugammadex use in the ED for neurologic exam is overall rare and appears most prevalent at academic medical centers. This likely reflects the capacity for advanced therapeutics and neurosurgical intervention for which rapid NMB reversal for neurologic exam is indicated at these centers and that adverse effects associated with its use are rare.^5^ ^,^ ^7^

## LIMITATIONS

A limitation of our study was lack of recorded TOF monitoring. Without TOF monitoring, it is difficult to comment on whether the sugammadex doses administered were adequate at fully reversing NMB. Because of the retrospective nature of the study, we were unable to determine the exact time of neurological examination. Additionally, it is possible that documented change in GCS was due to other factors such as changes in sedation or underlying neurologic status. An additional limitation of our study was that recorded GCS was based on exams performed by many different individuals with variable training backgrounds including nursing, emergency medicine residents and attendings, neurology and neurosurgery residents, and neurosurgery attendings. Further, the pre- and post-GCS was often based on examinations performed by separate individuals, and variation in exam between clinicians could have contributed to change in GCS, rather than true clinical change. Time of GCS examinations was also not recorded, which also may have affected the results of the exam after sugammadex administration. Although our cohort describes sugammadex use among patients with a range of neurological pathology, we did not capture any patients with status epilepticus as the underlying injury, one potentially relevant disease category for which NMB reversal has been previously described.^5^

An additional limitation was lack of bispectral index monitoring or data regarding awareness during paralysis. Awareness during paralysis is known to occur in ED patients receiving mechanical ventilation, with rocuronium being associated with increased frequency of awareness during paralysis.^21^ Furthermore, the impact of the use of sugammadex on clinical decision-making was difficult to determine given the retrospective nature of the study. Accurate neurological examination is an essential aspect of clinical decision-making during neurologic emergencies and likely played an important role in clinical course regardless of whether intervention was performed following repeat exam. Prospective research is needed to determine the impact of sugammadex on clinical decision-making.

Despite these limitations, sugammadex administration was well tolerated, with rare adverse effects. Although there were two episodes of hypotension and four episodes of bradycardia, it was difficult to determine whether these were attributable to sugammadex given possible confounders such as sedation administration and underlying critical illness. Overall, the cohort was associated with high acuity reflective of the critical nature of neurologic emergencies requiring intubation. Mortality was high in this cohort, and mRS at discharge was reflective of many patients having severe disability at discharge. Despite high mortality and severe disability in survivors, it is difficult to consider sugammadex administration and subsequent procedures as futile as some patients may go on to recover considerably with aggressive rehabilitation.

## CONCLUSION

Administration of sugammadex to facilitate neurologic examination is a rare occurrence in the ED. In this multicenter, retrospective study, we found that patients who received sugammadex in the ED during the study period had infrequent associated adverse effects, high rates of procedures within 24 hours of administration, and significant in-hospital mortality. Change in Glasgow Coma Scale was observed despite most patients in this cohort receiving sugammadex greater than one hour after NMB administration with a maximal observed interval of greater than four hours after NMB administration. Code status ultimately changed to “comfort measures only” for nearly half of these patients and, on average, patients discharged from a hospitalization where sugammadex had been administered in the ED had moderately severe neurologic disability. Prospective data is needed to assess the impact of sugammadex on decision-making.
